# Training Patterns and Mental Health of Bodybuilders and Fitness Athletes During the First Lockdown of the COVID-19 Pandemic—A Cross-Sectional Study

**DOI:** 10.3389/fspor.2022.867140

**Published:** 2022-05-03

**Authors:** Samuel Iff, Stefan Fröhlich, Robin Halioua, Christian Imboden, Jörg Spörri, Johannes Scherr, Ingo Butzke, Erich Seifritz, Malte Christian Claussen

**Affiliations:** ^1^Department of Psychiatry, Psychotherapy and Psychosomatics, Psychiatric University Hospital Zurich, University of Zurich, Zürich, Switzerland; ^2^University Center for Prevention and Sports Medicine, Department of Orthopaedics, Balgrist University Hospital, University of Zurich, Zürich, Switzerland; ^3^Sports Medical Research Group, Department of Orthopaedics, Balgrist University Hospital, University of Zurich, Zürich, Switzerland; ^4^Private Clinic Wyss AG, Münchenbuchsee, Switzerland; ^5^Psychiatriezentrum Münsingen (PZM) Centre for Psychiatry Münsingen, Münsingen, Switzerland; ^6^Adult Psychiatry, Psychiatric Services Grisons, Chur, Switzerland

**Keywords:** COVID-19, SARS-CoV-2, resistance training, bodybuilding, mental health, sports psychiatry

## Abstract

**Background:**

Government restrictions during the first COVID-19 lockdown, such as the closure of gyms and fitness centers, drastically limited the training opportunities of bodybuilders and fitness athletes (BoFA) who rely on indoor training facilities. This provided a unique situation to investigate the effect of training limitations on the training patterns, training adaptive strategies and mental health of BoFAs.

**Objectives:**

The primary aim of this study was to investigate differences in the training patterns and the mental health of BoFA before and during the first COVID-19 lockdown. The secondary aim was to assess whether BoFA who exhibited features of muscle dysmorphia were affected differently from the group that did not.

**Methods:**

A cross-sectional study was conducted with 85 BoFAs by means of an online questionnaire asking about sports activity, intensity, subjective physical performance, and economic status, including primary or secondary occupations before (from memory) and during lockdown, current physical health problems and financial fears, symptoms of depression, sleep disorders, anxiety (trait and state), muscle dysmorphia, coping mechanisms and actions during the first lockdown in Switzerland.

**Results:**

Training patterns and mental health of BoFA were influenced by the COVID-19 pandemic and first lockdown. During lockdown, the physical activity on the BoFA dropped significantly from 2.3 ± 0.8 h per day to 1.6 ± 0.9 h per day (*p* < 0.001), the subjective training intensity decreased significantly from 85.7 ± 13.2% to 58.3 ± 28.3% (*p* < 0.001) and the subjective performance declined significantly from 83.4 ± 14.3% to 58.2 ± 27.8% (*p* < 0.001) of maximal performance. In comparison to those without risk for body dysmorphia, participants at risk rated their maximal performance significantly lower and scored significantly higher for depression, sleep disorders and anxiety.

**Conclusion:**

This study showed the significant changes on the training patterns of BoFA before and during the first COVID-19 lockdown and poor mental health scores of BoFA during the lockdown itself, with those at risk of muscle dysmorphia scoring statistically worse regarding mental health than those with no risk of muscle dysmorphia. To better understand the particularities of BoFA, further investigation is needed to understand their psychology and in particular the effect of training restrictions on it.

## Introduction

The COVID-19 pandemic at the beginning of 2020 led to worldwide restrictions regarding public life and freedom of movement, with most countries going into lockdown at some point. Switzerland and Austria imposed a first lockdown on March 16, 2020 (Änderung, 2020), followed by Germany on March 22nd. This may have affected people's physical training opportunities, potentially affecting self-identified bodybuilders and fitness athletes (BoFA) on a motivational, emotional, and performance level. BoFA training schedules are based on rather inflexible gym-based training patterns and therefore have the potential to be especially affected by gym closures. Accordingly, we assume that this situation represented an enormous stress factor for bodybuilders and fitness athletes, similar to the general population (Hossain et al., 2020).

Very little is known about the importance of adherence to training schedules for BoFA regarding their mental wellbeing, and the 2020 lockdown restrictions serve as a potentially unique research opportunity. BoFA are known to have specific psychological traits and motivational patterns for achieving their goals, which can also result in higher rates of anxiety or other mental health problems (Schwerin et al., 1996; Steele et al., 2019). The potential obsessive-compulsive dimension of sports and exercise and, more specifically, addictive behavior should also be considered in this context (Weinstein and Weinstein, 2014). Disordered eating and muscle dysmorphia are likewise common mental health problems in fitness athletes and bodybuilder populations (Mitchell et al., 2017; Eichstadt et al., 2020; Neglia, 2021). Additionally, other motivational issues, such as the drive for leanness and muscularity, are important factors for their psychological wellbeing, and their wish to reshape the body to conform with aesthetic, hygienic, or sports norms (Coquet et al., 2018). For some of them, exercise also represents a form of coping with mental health problems (Rimer et al., 2012). Due to this unique mindset, which could lead to a higher vulnerability, and the BoFA's dependency on indoor training facilities, we hypothesize that altered training patterns, such as those induced by restrictions during the first COVID-19 lockdown, may also have had an influence on mental health variables.

The primary aim of this study was to investigate the differences in the training patterns of BoFA before and during the first COVID-19 lockdown and the mental health of BoFAs during lockdown itself.

The secondary aim was to assess whether BoFA who exhibited features of muscle dysmorphia were affected differently from the group that did not.

## Materials and Methods

### Study Design and Setting

The study was designed as a cross-sectional observation of self-identified bodybuilders and fitness athletes during the first lockdown as part of a larger project investigating mental health issues in high-performing sports populations during the COVID-19 pandemic (Fröhlich et al., 2021).

The first lockdown in Switzerland lasted 8 weeks from March 16 to May 11, 2020, and included the closure of many public places, including fitness clubs and gyms. Individual training indoors and outdoors was still possible. The restrictions applied to all participants of the present study. The participants were asked about their habits and circumstances before and during the lockdown. The study was approved by the local ethics committee by a declaration of non-responsibility (KEK-ZH-NR: Req-2020-00408). Participants or the public were not involved in the design, conduct, reporting, or dissemination of our research.

### Participants

This BoFA substudy was advertised on social media. Multiple BoFA with large numbers of followers were approached on Instagram and asked to post-details of the study. Participants clicked on a link within the Instagram posts and were taken to the study website. They were included in the study if they engaged in training or worked out at least 1 h per day on a regular basis before lockdown and self-identified themselves as either a bodybuilder or fitness athlete, defined as pursuing the sportive activity with mainly the goal of enhancing physique. Recruitment lasted 30 days and started on the 25th of April 2020 and ended on the 25th of May 2020, 2 weeks after ending many restrictions. Participants with incomplete data were excluded. Participants did not receive any compensation for participating.

### Data Collection and Evaluation

Data were collected with a REDCap-based online survey (Harris et al., 2019). Demographic data were collected, such as age (years), sex (m/f), work as a percentage of full time and whether income was generated from sports-related activities or other. Participants were asked to assess their training patterns with the following before (from memory) and during lockdown as training activity (hours/day), intensity, subjective maximal performance, and general financial fears, each rated on a scale of 0–100%. Intensity was defined as the self-perceived training intensity during a regular workout. Subjective maximal performance was defined as the self-perceived percentage of their own maximal athletic performance at the moment of questioning. Financial fears were the fears of losing their stream of income or of loss of financial security.

Anxiety ratings were obtained using the German version of the short form of the Spielberger State-Trait Anxiety Inventory (STAI) (Grimm, 2009). To investigate symptoms of depression, the 9-item depression module of the Patient Health Questionnaire (PHQ-9) was used (Kroenke et al., 2001). The Insomnia Severity Index (ISI) and extracts from the Pittsburgh Sleep Quality Index were used to assess any sleep disturbances (Bastien et al., 2001). Participants were asked how well they subjectively coped with the lockdown measures [visual analog scale (VAS) 0–100] and whether they were worried about continuing their sports careers (VAS scale, 0 = coping badly to 100 = coping well). To adjust for current sports-related health problems such as traumatic injuries, overuse injuries or illnesses, the Oslo Sports Trauma Research Center (OSTRC) questionnaire on health problems was used (Clarsen et al., 2013). History of SARS-CoV-2 and/or quarantine periods was also collected.

To understand the mechanisms of how BoFA adapted to the lockdown, participants were asked additional questions (*via* a follow survey link sent by email) involving what they did in detail to compensate for the closed facilities during lockdown. These questions included how they continued to work out, if and how much they invested in a home gym, how well they were subjectively coping with the closure of gyms (VAS, 0 = coping badly to 100 = coping well), if they were afraid of losing muscle or gaining fat and if they were adapting their nutrition (VAS 0 = not at all to 100 = entirely). Additionally, participants were invited to complete the Muscle Dysmorphic Disorder Inventory (MDDI) (Zeeck et al., 2018). An MDDI score ≥39 indicates a risk for muscle dysmorphia (Varangis et al., 2012).

### Statistical Methods

Baseline data are expressed as the mean ± standard deviation (SD) and frequency tables for categorical data. BoFA subjective athletic performance, training activity and intensity were compared before and during lockdown. Comparisons of data between groups were performed using the two-tailed independent sample *t-*test for continuous data and χ^2^-tests for categorical data. In tables with more than two groups, the χ^2^-test was used for tables with more than four fields, one-way analysis of variance was used for continuous variables, and the Kruskal–Wallis rank test was used for non-metric variables. A two-sided *p* < 0.05 was considered statistically significant.

For investigating the effects of government restrictions on the three variables activity, maximal subjective performance and intensity, we fitted a general linear model (GLM) using value of activity, maximal subjective performance and intensity during the government restrictions as the dependent variable, retrospective variable from memory as the independent variable and age, gender, injury, coping with protective measures, occupation percentage and sufficient income before the restrictions as covariates. To adjust for mental influences, we added the individual sums of PHQ9, ISI, TRAIT and STATE anxiety, MDDI as well financial fears and worries of career. All explanatory variables that had an association with performance at *p* < 0.20 in the univariable analyses were included in the multivariable-adjusted analyses. Using a stepwise backward elimination process, the least significant variables were then removed from the base model. Only variables with *p* < 0.05 remained in the final parsimonious model.

Stata Statistical Software: Release 13. College Station, TX, was used to analyze the data.

## Results

### Response Rate

Overall, 85 BoFA were eligible and included in the study. The results of the larger study targeting elite athletes as a cohort with a substantially different background and context have been published elsewhere (Fröhlich et al., 2021). Of the 85 BoFAs, 47 (55%) replied to the additional survey and allowed more detailed insight into their adaptions and coping behavior during lockdown and their MDDI scores.

### Baseline Data

Baseline data are shown in Table 1. Thirty-three (39%) BoFAs replied during the lockdown, 52 (61%) shortly after the lockdown where some facilities were opened again. Most BoFAs were male (*n* = 75, 86%), and the mean (±SD) age was 26.5 ± 7.5 years. During the lockdown, 77% of the BoFAs generated income from non-fitness related activities, and from the remaining 23%, 32% generated sufficient income from their sporting activity alone. However, many of them were worried about their sports careers because of the lockdown, and financial fears were significantly higher during lockdown than before lockdown (21.6 ± 28.4 vs. 11.3 ± 17.9, *p* = 0.005). None of the athletes had a positive SARS-CoV-2 test, but 5 people had to quarantine for being in contact with a positive person.

**Table 1:** Data of bodybuilders and fitness athletes (BoFA) during lockdown (*N* = 87).

	**During lockdown**
**Baseline data**	
Female gender	*n* = 12, (14%)
Age at survey date	26.5 ± 7.5
Additional occupation in %	77 ± 25
**Financial situation**	
Sufficient from sportive activity	32%
Financial fears (0–100)	21.6 ± 28.4
Worried for sportive career (0–100)	49.4 ± 34.1
**COVID-19 related data**	
Positive SARS-CoV-2 test	0 (0%)
Quarantine (yes)	5 (6%)
Quarantine (days)	12.2 ± 6.3
Coping with lockdown	57.0 ± 30.6
**Physical activity data**	
Activity (hours/day)	1.6 ± 0.9
Intensity (%)	58.1 ± 29.0
Max. subjective performance (%)	58.2 ± 28.2
Effective sleep time (hours)	7.1 ± 1.2
Self-reported injury/illness (yes)	*n* = 25 (29%)
**Psychiatric measures**	
ISI sum (0–28)	9.0 ± 5.8
PHQ9 sum (0–27)	9.6 ± 5.5
STAI trait sum (20–80)	35.9 ± 12.1
STAI state sum (20–80)	37.8 ± 13.5
MDDI sum (13–65)	39.9 ± 7.1

Spielberger State-Trait Anxiety Inventory (STAI), Patient Health Questionnaire (PHQ-9), Insomnia Severity Index (ISI), Muscle Dysmorphic Disorder Inventory (MDDI), Numbers are means ± standard deviation. Percentages in brackets are the proportion of the total.

### Changes From Pre-lockdown to During the Lockdown

As shown in Table 2, during lockdown, the physical activity of the BoFA dropped significantly from 2.3 ± 0.8 h per day to 1.6 ± 0.9 h per day (*p* < 0.001), the subjective training intensity decreased significantly from 85.7 ± 13.2% to 58.3 ± 28.3% (*p* < 0.001) and the subjective performance declined significantly from 83.4 ± 14.3% to 58.2 ± 27.8% (*p* < 0.001) of maximal subjective performance. Financial fears increased significantly from 11.5 ± 18 (on a scale from 0 to 100) before lockdown to 21.2 ± 28.1 during the lockdown (*p* = 0.005). In the ISI, the mean score was 9.1 ± 5.9, the PHQ9 sum mean was 9.4 ± 5.5, the mean trait anxiety was 35.4 ± 11.5, and the mean state anxiety was 37.3 ± 13.3.

**Table 2:** Data of bodybuilders and fitness athletes (BoFA) before lockdown (*N* = 87).

	**Before lockdown (from memory)**	**During lockdown**	***p*-value for difference**
**Physical activity data**			
Activity (hours/day)	2.3 ± 0.8	1.6 ± 0.9	<0.001
Intensity (%)	85.7 ± 13.2	58.1 ± 29.0	<0.001
Max. subjective performance (%)	83.4 ± 14.3	58.2 ± 28.2	<0.001
**Financial situation**			
Financial fears (0–100)	11.5 ± 18.0	21.6 ± 28.4	0.005

In comparison to pre-lockdown values, 9 (10%) participants increased their activity by at least 20%, 49 (56%) decreased their activity by at least 20%. Five (6%) participants increased their subjective performance by at least 20%, 51 (59%) decreased their subjective performance by at least 20%. Four (5%) participants increased their maximal subjective performance by at least 20%, 49 (56%) decreased their maximal subjective performance by at least 20%.

Additional questions were answered by 44 participants. The baseline data were not significantly different between those who replied to the additional questionnaire and those who did not. Of the 44 participants who answered the additional questionnaire (how they reacted to the lockdown), 5 stopped having access to a gym, 19 used other private gyms, 23 continued to train with bodyweight, and 14 bought their own home gym equipment. The 14 who bought equipment invested 494.7 ± 455.5 Swiss Francs (CHF) during the lockdown. Not coping with restrictions was rated 57.0 ± 37.1 on the VAS. In this group, the fear of losing muscle was rated to be 43.5 ± 39.8, the fear of gaining fat was 47.0 ± 37.2 and the likelihood of adapting nutrition was 54.4 ± 36.5.

The MDDI was 39.9 ± 7.2. An MDDI score ≥39 indicates a risk for muscle dysmorphia. Participants at risk for muscle dysmorphia rated their maximal subjective performance significantly lower, scored significantly higher for all four psychometric scores and indicated significantly more problems with coping with gym closure (Table 3).

**Table 3:** Comparison between participants at muscle dysmorphia risk.

	**MDDI <39**	**MDDI ≥39**	
	***N* = 23**	***N* = 21**	***p*-value**
**Baseline data**			
Age at survey date	25.4 ± 9.3	27.0 ± 5.9	n.s.
Female gender	*n* = 3 (13%)	*n* = 5 (24%)	n.s.
**COVID-19 related data**			
Coping with protective measures	61.7 ± 28.3	48.5 ± 34.5	n.s.
**Physical activity data**			
Activity (hours/day)	1.6 ± 0.9	1.5 ± 0.7	n.s.
Intensity (%)	67.4 ± 27.9	53.7 ± 24.7	n.s.
Max. subjective performance (%)	67.6 ± 24.0	51.7 ± 24.3	0.035
Effective sleeping time (hours)	8.3 ± 1.0	8.2 ± 1.0	n.s.
Self-reported injury/illness (yes)	*n* = 9 (39%)	*n* = 8 (38%)	n.s.
**Psychiatric measures**			
ISI sum (0–28)	6.7 ± 5.1	10.3 ± 5.6	0.032
PHQ9 sum (0–27)	6.5 ± 4.1	11.0 ± 5.3	0.003
TRAIT sum (20–80)	30.1 ± 11.2	40.8 ± 9.5	0.002
STATE sum (20–80)	32.2 ± 10.9	43.0 ± 13.7	0.006
**Fitness lockdown related questions**			
No gym (yes)	3 (13%)	2 (10%)	n.s.
Use other private gym (yes)	11 (48%)	8 (38%)	n.s.
Train with bodyweight (yes)	10 (43%)	13 (62%)	n.s.
Buy own gym (yes)	8 (35%)	8 (38%)	n.s.
Not coping with gym closure (0–100)	45.6 ± 37.9	69.4 ± 32.6	0.032
Money spent (CHF)	696.2 ± 602.0	298.8 ± 145.4	n.s.
Fear of losing muscle (0–100)	53.2 ± 39.7	32.8 ± 38.0	n.s.
Fear of gaining fat (0–100)	56.4 ± 35.4	36.6 ± 37.1	n.s.
Adapting nutrition (0–100)	58.1 ± 36.5	50.4 ± 37.0	n.s.
MDDI sum	34.3 ± 4.2	46.0 ± 4.1	<0.001

Spielberger State-Trait Anxiety Inventory (STAI), Patient Health Questionnaire (PHQ-9), Insomnia Severity Index (ISI), Muscle Dysmorphic Disorder Inventory (MDDI). Numbers are means ± standard deviation. Percentages in brackets are the proportion of the total answers.

The results of the GLM multivariate regression for the effect of government restrictions on activity, intensity and maximal subjective performance is shown in Table 4. Activity was only significantly influenced by the STATE sum (b-coefficient 0.040), Intensity didn't show any significant influencing variables and maximal subjective performance was increased significantly by the maximal subjective performance before lockdown (b-coefficient 1.1158) and TRAIT sum (b-coefficient 0.882), and reduced by PHQ9 sum (b-coefficient −2.664).

**Table 4:** Multivariate regression of activity, intensity and maximal subjective performance during lockdown.

	**Activity**	**Intensity**	**Max. subjective**
	**(hours/day)**	**(%)**	**performance (%)**
	***b***	***b***	***b***
Activity before	0.115	-	-
(hours/day)			
Intensity before (%)	-	*p* > 0.2	-
Max. subjective	-	-	1.158^***^
performance			
before (%)			
ISI sum	0.034		
PHQ9 sum	−0.075	−1.595	−2.664^**^
TRAIT sum	−0.022		0.882^*^
STATE sum	0.040^*^	0.731	
MDDI	−0.009	−1.035	−0.707
constant	1.054	71.040^**^	−23.388

* *p < 0.05*.

** *p < 0.01*.

*** *p < 0.001*.

All models were fully corrected for age, gender, injury, coping with protective measures, occupation percentage, sufficient income before the restrictions, previous levels of activity, maximal subjective performance and intensity, respectively.

## Discussion

The major findings of this study were: (1) physical activity dropped significantly in comparison with pre-lockdown levels measured as workout intensity, performance, and activity; (2) signs of insomnia, depression, and anxiety were high in BoFa during lockdown; and (3) in comparison to participants without risk for muscle dysmorphia, those at risk for muscle dysmorphia rated their maximal performance significantly lower and scored significantly higher in PHQ-9, ISI, and STAI.

The physical activity, subjective training intensity and subjective performance of BoFA dropped significantly during lockdown. The drop from pre-lockdown values in physical activity, subjective training intensity and performance is plausible and is in line with other research that has been published on the effects of the lockdown on this particular population (Zoob Carter et al., 2021). This represents a pattern similar to other studies, which reported a reduced training frequency, shorter training sessions and lower sport-specific intensity in many other sports (Washif et al., 2021). However, 10% BoFA increased their activity as a possible answer to the increased stress they were experiencing, which is in line with previously published research (Stults-Kolehmainen and Sinha, 2014).

In our sample of BoFA, financial fears were significantly higher during the lockdown. The reduction of sportive activity could have triggered this additional fear. The 32% of BoFAs whose income was sufficient from their sporting activity was surprisingly high and could be due to a selection bias generated by recruitment through social media. Social media itself could be the source of income through, for example, sporting sponsorship deals. This could also explain why roughly half of the participants were worried about their sports careers due to the lockdown, as they feared the loss of their ability to train, present their physique online and not generate enough income. Additionally, BoFAs require an intense regimen of weightlifting and dieting culminating in an on-stage posing competition, especially if they are visible on social media. This drop in performance could increase or trigger psychopathologies since BoFAs are known to have an increased risk for four categories of psychopathology: muscle dysmorphia, disordered eating, use of image and performance enhancing drugs, and exercise dependence (Schwerin et al., 1996). This finding is reflected in all four measured psychological dimensions (PHQ9, ISI, trait and state anxiety), where the BoFA scored highly. They expressed elevated values for ISI—despite not above the clinical cut-off -, meaning their sleep was less restful, and higher values in PHQ9, suggesting moderate signs of depression, indicating that they are at higher risk for depressive disorders. The higher prevalence of symptoms in the BoFA is well in line with the general traits that have been found in other studies where higher traits of social anxiety were found (Schwerin et al., 1996).

On the other hand, physical activity has a well-known protective and therapeutic effect against the development of psychiatric symptoms such as anxiety, depression, and sleep disturbances (Kredlow et al., 2015; Stonerock et al., 2015; Imboden et al., 2021); the loss of this protective effect could explain the high scores. In addition, for those with an element of exercise addiction or dependence, the direct psychological effect of the inability to train alone could explain the high scores, or of course a combination of both. Our data does not allow us to attribute one mechanism over the other.

In accordance with trait and state anxiety, the BoFA rated their fear of losing muscle or gaining fat during the lockdown as rather high (~50 points out of 100 each). Taking into consideration that the mean MDDI was 39.9, we can assume that some BoFAs were at risk for muscle dysmorphia (MD) (Zeeck et al., 2018). We assume that the lockdown may had also influenced eating habits and weight-lifting routines, each of which may have influenced the risk of MD behavior. Because the gyms were closed, alternative training options had to be found, which may not provide the same training stimulus as before and may therefore trigger the fear of muscle loss. Additionally, discontinuation of previous daily structures may have led to an involuntary change in eating behavior, including increased snacking and grazing (Rodgers et al., 2020), and therefore triggered the fear of fat gain. These alterations in eating and training habits equally affect healthy BoFAs and those at risk for MD. However, due to heightened muscularity and leanness concerns of those at high risk for MD, it can be assumed that these changes are less easily compensated for, leading to increased anxiety.

Despite the lockdown, 9 out of 10 BoFA continued their workouts with either home gym, body weight or other private gyms. The amount of money spent buying equipment was ~500 CHF with large variability. The maximum spent was above 2,000 CHF, suggesting that this BoFA bought a full home gym set-up. Some participants may have already had equipment at home and did not need to invest in any new, couldn't afford it due to high demand and rapidly escalating prices, or could use another private gym instead where perhaps only minor investments were necessary.

In the comparison between BoFAs at risk for MD compared to those without risk, there was a significant difference in the subjective maximal performance. This suggests that confidence of BoFA at risk for MD in their performance dropped more quickly during the lockdown. The fact that athletes at risk for MD scores did not differ regarding compensatory measures (buying equipment, etc.) despite worse scores in anxiety and subjective performance is unexpected.

In Table 4, MDDI was not significant in the final parsimonious model on the multivariable model of effects on activity, intensity or maximal performance. Depressive symptoms and trait anxiety had a significant influence on maximal subjective performance. This suggests that depressive symptoms had an influence of the perceived performance of the BoFA. Activity was only influenced significantly by state anxiety, but not by depressive symptoms. Based on the extended analysis shown in Table 4, there was no sign of influence of depressive symptoms measured by PHQ9 on activity or intensity, but a significant negative effect on subjective maximal performance.

### Limitations

Due to the unexpected pandemic, we were only able to gather data about the pre-pandemic situation from memory of the participants (retrospectively). There could be a recall bias about the situation before the lockdown and the data collected (like training intensity and frequency) could have been different from reality. Also, the cross-sectional design of the study does not allow any interpretations regarding cause-effect relationship of the lockdown restrictions. In addition, mental health tools are validated for current psychological status only and so we were unable to do a comparison in mental health pre and during lockdown, our study gives only a snapshot of the mental health of BoFA during lockdown. Another important limitation may be considered the recruitment method resulting in a potentially non-representative sample. The data collection of this convenience sample through social media represents an important selection bias where the people in the fitness group were searched through social media, leaving out athletes with less or no social media activity. Hence, the data need to be generalized very cautiously. This study looked at a subsample of athletes during very particular conditions. The data indicate that the athletes were influenced by the lockdown. However, in a complex and multifactorial system of determinants underlying mental health, a certain risk of bias from unknown confounders may remain, which finally limits the possibility of drawing conclusions of cause and effect. Also, the study was not powered for a comparison between BoFA at risk for MD and those without risk. These results must be interpreted with caution. Finally, there were many different factors at play as stressors in the special situation of the lockdown, including amongst others, the pandemic itself, the decreased access to gyms, changes in exercise routine and this study cannot untangle the effects specific to each.

## Conclusion

The lockdown of spring 2020 during the pandemic provided an ideal and potentially unique opportunity to investigate the effects of restricted training opportunities in BoFA.

This study highlights the change in training patterns of BoFA before and during the lockdown. Physical activity, training intensity as well as perceived sports performance dropped significantly in comparison with pre-lockdown levels. During the lockdown BoFA scored highly on insomnia, depression and anxiety scores, suggesting effects on mental health. BoFA at risk for muscle dysmorphia scored statistically higher for these mental health indicators when compared to the group without. To better understand the particularities of BoFA, further investigation is needed to understand their psychology and in particular the effect of training restrictions on it.

## Data Availability Statement

The raw data supporting the conclusions of this article will be made available by the authors, without undue reservation.

## Ethics Statement

The studies involving human participants were reviewed and approved by Kantonale Ethik Kommission Zürich. The patients/participants provided their written informed consent to participate in this study.

## Author Contributions

MC, JSp, JSc, and SF conceptualized and designed the study. Additional questions for bodybuilders and fitness athletes were designed by RH in discussions with the MC and SF. SF and MC recruited the participants, collected the data, and drafted the current manuscript. SI processed the data and performed the statistical analysis. All authors substantially contributed to the interpretation of the data, revised it critically, approved the final version of the manuscript, and agreed to be accountable for all aspects of the work.

## Conflict of Interest

The authors declare that the research was conducted in the absence of any commercial or financial relationships that could be construed as a potential conflict of interest.

## Publisher's Note

All claims expressed in this article are solely those of the authors and do not necessarily represent those of their affiliated organizations, or those of the publisher, the editors and the reviewers. Any product that may be evaluated in this article, or claim that may be made by its manufacturer, is not guaranteed or endorsed by the publisher.
